# ‘Proteolytic switching’: opposite patterns of regulation of gelatinase B and its inhibitor TIMP-1 during human melanoma progression and consequences of gelatinase B overexpression

**DOI:** 10.1038/sj.bjc.6690385

**Published:** 1999-05-01

**Authors:** J R MacDougall, M R Bani, Y Lin, R J Muschel, R S Kerbel

**Affiliations:** Cancer Biology Research, Sunnybrook Health Science Centre, 2075 Bayview Avenue, Toronto, Ontario, Canada M4N 3M5; Department of Medical Biophysics, University of Toronto, Toronto, Ontario, Canada; Pathology and Laboratory Medicine, University of Pennsylvania School of Medicine, Philadelphia, PA 19104, USA

**Keywords:** gelatinase B, TIMP-1, cytokines, tumour progression, melanoma

## Abstract

Although it is generally accepted that proteolytic degradation is an important mechanism used by malignant cells in the process of metastasis, comparatively little is known about the regulation of molecules responsible for proteolysis and how they become de-regulated during human tumour progression. Using a genetically related pair of human melanoma cell lines, derived from the same patient at different stages of disease, we analysed differences in the cytokine-mediated regulation of gelatinase B (MMP-9), an enzyme thought to play an important role in cellular invasiveness, and TIMP-1, a physiological inhibitor of this enzyme. Whereas the advanced stage (i.e. metastatic) partner of this pair (WM 239) could produce gelatinase B upon induction with interleukin (IL)-1β or tumour necrosis factor alpha (TNF-α), the early stage (i.e. primary) partner (WM 115) could not. In sharp contrast, we found that TIMP-1 displayed an opposite pattern of induction in these cell lines. Specifically, the early stage cell line, WM 115, demonstrated a marked increase in the production of TIMP-1 when treated with IL-1β or TNF-α whereas the advanced cell line, WM 239, showed no such increase. Treatment with the DNA demethylating agent, 2-deoxy-5-azacytidine, resulted in a marked up-regulation of both gelatinase B and TIMP-1 in both cell lines. It was further found that constitutive overexpression of gelatinase B in WM 239 cells and an additional melanoma cell line (MeWo), derived from a metastatic lesion, was able to greatly enhance lung colonization in an experimental metastasis assay while we did not observe differences in tumorigenicity. From these results we conclude that an altered responsiveness of gelatinase B and TIMP-1 to induction by similar agents is associated with disease progression in human melanoma and that this altered responsiveness can have consequences to the aggressive nature of the disease. © 1999 Cancer Research Campaign


*British Journal of Cancer* (1999) **80**(3/4), 504–512
© 1999 Cancer Research Campaign
Article no. bjoc.1998.0385

### ‘Proteolytic switching’: opposite patterns of regulation of gelatinase B and its inhibitor TIMP-1 during human melanoma progression and consequences of gelatinase B overexpression

**JR MacDougall[1,2], MR Bani[1]*, Y Lin[1], RJ Muschel[3] and RS Kerbel[1,2]**

[1]Cancer Biology Research, Sunnybrook Health Science Centre, 2075 Bayview Avenue, Toronto, Ontario, Canada M4N 3M5; [2]Department of Medical Biophysics, University of Toronto, Toronto, Ontario, Canada; [3]Pathology and Laboratory Medicine, University of Pennsylvania School of Medicine, Philadelphia, PA 19104, USA

**Summary** Although it is generally accepted that proteolytic degradation is an important mechanism used by malignant cells in the process of metastasis, comparatively little is known about the regulation of molecules responsible for proteolysis and how they become de-regulated during human tumour progression. Using a genetically related pair of human melanoma cell lines, derived from the same patient at different stages of disease, we analysed differences in the cytokine-mediated regulation of gelatinase B (MMP-9), an enzyme thought to play an important role in cellular invasiveness, and TIMP-1, a physiological inhibitor of this enzyme. Whereas the advanced stage (i.e. metastatic) partner of this pair (WM 239) could produce gelatinase B upon induction with interleukin (IL)-1β or tumour necrosis factor alpha (TNF-α), the early stage (i.e. primary) partner (WM 115) could not. In sharp contrast, we found that TIMP-1 displayed an opposite pattern of induction in these cell lines. Specifically, the early stage cell line, WM 115, demonstrated a marked increase in the production of TIMP-1 when treated with IL-1β or TNF-α whereas the advanced cell line, WM 239, showed no such increase. Treatment with the DNA demethylating agent, 2-deoxy-5-azacytidine, resulted in a marked up-regulation of both gelatinase B and TIMP-1 in both cell lines. It was further found that constitutive overexpression of gelatinase B in WM 239 cells and an additional melanoma cell line (MeWo), derived from a metastatic lesion, was able to greatly enhance lung colonization in an experimental metastasis assay while we did not observe differences in tumorigenicity. From these results we conclude that an altered responsiveness of gelatinase B and TIMP-1 to induction by similar agents is associated with disease progression in human melanoma and that this altered responsiveness can have consequences to the aggressive nature of the disease.

**Keywords:** gelatinase B; TIMP-1; cytokines; tumour progression; melanoma

Tumour progression is associated with the accumulation of genetic changes which can result in eventual acquisition of malignant properties, i.e. in the emergence of tumour cell populations that have acquired a sufficient number of properties to allow them to metastasize. In this regard, proteolytic degradation of the extracellular matrix is thought to play a crucial role in the liberation and seeding of cancer cells during the process of metastasis (Stetler-Stevenson et al, 1996). Furthermore, the family of matrix metalloproteinases (MMP) is likely to be responsible for much of the proteolytic degradation associated with tumour cell invasion. This is because members of this family have a wide substrate specificity, including type IV or basement membrane collagen, and are often associated with normal and disease processes in which turnover of matrix components is essential to the process (MacDougall and Matrisian, 1995). A role for the MMPs in metastasis is also supported by the fact that transfection of metastatic cancer cell populations with the gene that encodes the endogenous inhibitor of MMPs, tissue inhibitor of metalloproteinase (TIMP-1), markedly inhibits both experimental and spontaneous metastasis in a number of models (DeClerck et al, 1992). This regulatory role of TIMPs suggests that it is the net proteolytic balance between the production of enzymes and their inhibitors that determines the metastatic capacity of the cells (DeClerck and Imren, 1994).

The MMP subfamily of the gelatinases, in particular, has been a focus of study since these enzymes have been shown to have the ability to degrade type IV collagen (Collier et al, 1988; Wilhelm et al, 1989), a property thought to be required by all cells crossing any basement membrane. Gelatinase B has been demonstrated, by transfection analysis, to confer metastatic competence upon non-metastatic rat fibroblastic cells, thus implicating it as a possible key enzyme in the process of metastasis (Bernhard et al, 1994). The demonstration that gelatinase B is involved in tissue invasion of normal trophoblasts (Librach et al, 1991) and macrophages (Watanabe et al, 1993) also lends support to its role as an enzyme mediating invasiveness of both normal and malignant cells. Despite this information, however, little is known about the regulation of gelatinase B expression, and how it can become de-regulated during cancer progression. Studies have shown the gene which

*Received 23 June 1998*
*Revised 29 October 1998*
*Accepted 11 November 1998*

*Correspondence to:* JR MacDougall, Cancer Research Programme, Northwestern Ontario Regional Cancer Centre, 290 Munro Street, Thunder Bay, Ontario, Canada P7A 7T1

*Present address: Mario Negri Institute for Pharmacological Research, Bergamo 24100, Italy encodes gelatinase B is positively controlled by inflammatory cytokines such as tumour necrosis factor (TNF)-α and interleukin (IL)-1β (Sato and Seiki, 1993; Unemori et al, 1991), growth factors such as transforming growth factor (TGF)-β (Salo et al, 1991) and other agents such as TPA (Moll et al, 1990). Gelatinase B expression has also been shown to be negatively controlled by the adenovirus E1A protein (Frisch et al, 1990) and as a consequence of somatic fusion in melanoma cells (MacDougall et al, 1995).

Regulation of MMPs by cytokines and other agents is only one of the ways in which the net proteolytic balance of tumour cells can be tipped in favour of matrix degradation. Thus, the inhibitors of MMPs are also thought to contribute an important role in determining the extracellular activity of secreted MMPs. The TIMP family, which consists of four members, TIMP-1, -2, -3 and -4, are thought to function by binding to the active site of matrix metalloproteinases, and hence inhibiting their function. Like the MMPs, the TIMPs also have been found to be regulated by a number of different cytokines and growth factors. Perhaps most significantly, TGF-β has been demonstrated to play a pivotal role in the regulation of wound healing (Mustoe et al, 1987), possibly by induction of cellular TIMP production (Edwards et al, 1987). Inflammatory mediators such as IL-1β and TNF-α have also been shown to regulate the levels of TIMPs produced by various cell types (Ito et al, 1990).

We have previously demonstrated that melanoma cells may undergo a 'switch' in permissivity of gelatinase B expression, that is, advanced melanoma cells are able to express gelatinase B either constitutively or through induction with cytokines while early stage melanoma cells do not express this molecule under any conditions (MacDougall et al, 1995). Using a genetically related pair of human melanoma cell lines, one derived from a primary melanoma, the other derived from a lymph node metastasis isolated 16 months later from the same patient (Kath et al, 1991), we have addressed the hypothesis that melanoma cells may undergo a 'proteolytic switch' during disease progression. As a consequence of this switch, we show that the more aggressive cells not only acquire the ability to produce gelatinase B in response to cytokine treatment but also lose the ability to produce an inhibitor of gelatinase B, specifically TIMP-1, after identical treatment. In contrast, the less aggressive cells are unable to produce gelatinase B under cytokine treatment, but maintain a robust TIMP-1 induction under similar circumstances. In addition, we describe a heretofore unreported effect of the DNA demethylating agent 2-deoxy-5-azacytidine on the production of gelatinase B and TIMP-1. We finally present evidence for a functional consequence of this 'proteolytic switch' by demonstrating a marked increase in lung colonization by melanoma cells engineered to constitutively express gelatinase B.

### MATERIALS AND METHODS

### Cell culture, collection of conditioned media and growth factors

Melanoma cell lines, kindly provided by Dr M Herlyn (Wistar Institute, Philadelphia, PA, USA) were routinely grown in RPMI-1640 (Gibco) media supplemented with 5% fetal bovine serum (FBS; Hyclone) in a humidified atmosphere containing 5% carbon dioxide at a constant temperature of 37°C. The cell lines were derived from the same patient in a sequential manner. WM 115 was derived from a vertical growth phase (VGP) primary lesion whereas WM 239 was derived from a lymph node metastasis 16 months later (Kath et al, 1991).

Conditioned media from the cell lines was collected as follows. Melanoma cells were plated at a density of $3.0 \times 10^5$ per well of a 6-well plate (Nunc) and incubated overnight in ExCell 300 media (JR Scientific) supplemented with 2% FBS. Cells were then washed with serum-free ExCell 300, incubated for 4 h and washed again with fresh serum-free ExCell 300 with or without TNF-α or IL-1β (UBI, Lake Placid, NY, USA) at a concentration of 5.0 ng $ml^{-1}$. Treatment with 2-deoxy-5-azacytidine followed a slightly different protocol. Cells were treated with 4.5 μM 2-deoxy-5-azacytidine for 48 h in serum containing medium prior to plating at a density of $3.0 \times 10^5$ per well of a 6-well plate and subsequent washes with serum-free medium as described above. In either case, after 48 h serum-free conditioned medium was centrifuged to remove cellular debris, aliquoted and either used immediately, or frozen at − 70°C.

### Quantitation of protein in melanoma cell conditioned medium

The concentration of protein secreted by melanoma cells into serum-free media was assayed by the method described by Bradford (1976). Briefly, 50 μl of serum-free conditioned media were aliquoted into the wells of a 96-well enzyme-linked immunosorbent assay (ELISA) plate and mixed 1:4 with a solution of 0.01% Coomassie brilliant blue G-250 (Merck) in 4.7% ethanol and 8.5% phosphoric acid (Sigma). After 5–10 min of incubation at ambient temperature, the absorbance of the samples were measured at 595 nm and corrected against 410 nm. Concentration of protein was calculated by substitution of values into a least squares fitted standard curve of bovine serum albumin (BSA; Pierce) between 0.5 and 10 μg $ml^{-1}$. All samples and standards were performed in quadruplicate and all sample points fell within the range of the standard curve.

### Substrate-embedded gel electrophoresis

Substrate-embedded gel electrophoresis, or zymography, was performed essentially as reported (Herron et al, 1986). Briefly, sodium dodecyl sulphate-polyacrylamide gel electrophoresis (SDS-PAGE) gels were prepared at a concentration of 10% containing 1.0 mg $ml^{-1}$ of gelatin (Sigma). The resolving gel was overlaid with a stacking gel of 5% acrylamide not containing gelatin. A volume of conditioned media equivalent to 200 ng of protein was mixed with SDS sample buffer without reducing agent or boiling and subsequently electrophoresed at a constant current of 20 mA per gel. Upon completion of electrophoresis the gels were washed twice for 15 min in 2.5% Triton X-100 (Sigma), rinsed briefly with water and then incubated overnight at 37°C in a solution of 50 mM Tris–HCl (Sigma), pH 7.0, 5 mM calcium chloride (Sigma) and 0.02% $NaN_3$ (Sigma). After incubation, the gels were stained in a solution of 0.1% Coomassie brilliant blue R-250 in 40% methanol and 20% acetic acid and destained in the same solution without Coomassie brilliant blue R-250. The resulting gels showed areas of clearing where the substrate within the gel has been digested away.

### Reverse transcription-polymerase chain reaction analysis

Messenger RNA for gelatinase B was assayed by reverse transcription-polymerase chain reaction analysis (RT-PCR) briefly as follows. RNA from control or treated cells was isolated by cell

© Cancer Research Campaign 1999

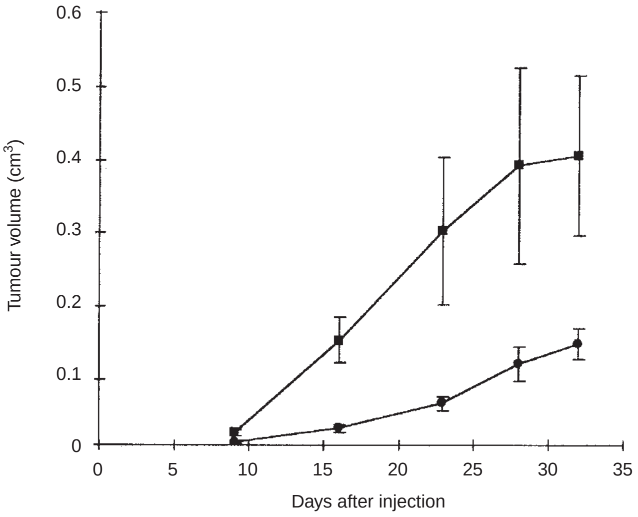

**Figure 1** Growth of WM 115 and WM 239 in vivo. One million WM 115 (●) or WM 239 (■) cells in 50 µl were injected subdermally into the flank of 6- to 8-week-old female nude mice. At the appearance of tumour growth, measurements and calculations were made as described in Materials and Methods. The data are representative and are presented as the mean ± s.d. of four animals for both WM 115 and WM 239

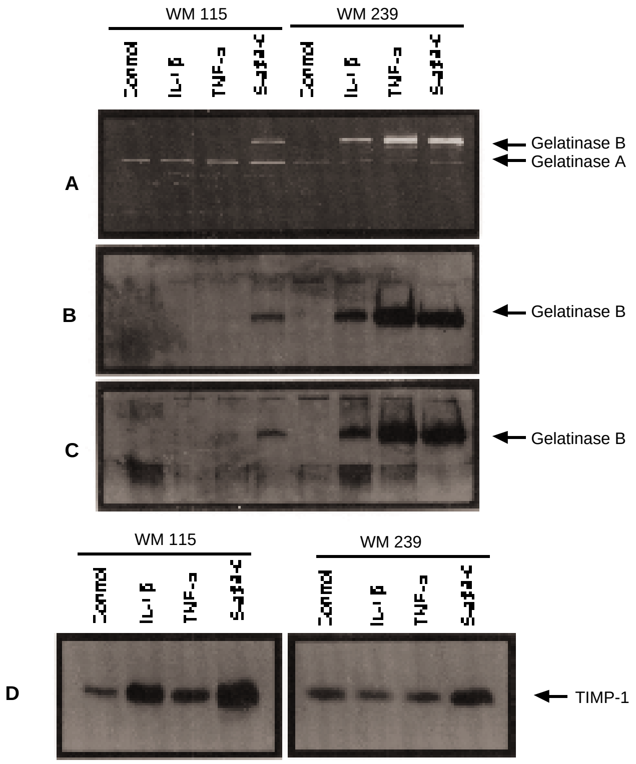

**Figure 2** Gelatin zymography and Western blot analysis of conditioned media and lysates from melanoma cell lines for the expression of gelatinase B and TIMP-1. WM 115 and WM 239 cells either untreated (control) or treated with 5.0 ng ml$^{-1}$ of IL-1β, 5.0 ng ml$^{-1}$ TNF-α or 4.5 µM 2-deoxy-5-azacytidine (5-aza-c). (**A**) Gelatin zymography of conditioned media from cells treated under the above conditions. The arrows indicate the migration of gelatinase A and B. Note no change in gelatinase A under all conditions. (**B**) A Western blot analysis of concentrated conditioned media (10 µg of protein) from cells treated under the above conditions. The arrow indicates the migration of gelatinase B. Note the similar pattern of production as seen with gelatin zymography. (**C**) Gelatinase B Western blot analysis of partially purified lysates from cells treated under the conditions described. (**D**) TIMP-1 Western blot analysis of 10 µg of protein from WM 115 and WM 239 cells treated under the conditions described lysis in 4.0 M guanidine isothiocyanate and subsequent phenol–chloroform extraction as previously described (Chomczynski and Sacchi, 1987). One µg of total RNA was reverse transcribed in a 20 µl reaction containing 200 units of Mu-MLV reverse transcriptase (Gibco), 1.0 mM each dNTP, 50 mM Tris–HCl pH 8.3, 75 mM potassium chloride (KCl), 10 mM dithiotreitol (DTT), 3.0 mM magnesium chloride ($MgCl_2$) and 0.1 µg oligo-$dT_{12}$. The products of reverse transcription were then subjected to 35 cycles of PCR in 10 mM Tris–HCl pH 9.0, 50 mM KCl, 1.5 mM $MgCl_2$, 0.5 units *Taq* polymerase (Pharmacia) and 50 pMol of each upstream and downstream primer. The cycle parameters were: 5 cycles of 5 min at 95°C, 3 min at 58°C and 5 min at 72°C, the remaining 30 cycles being performed for 1.5 min at each temperature. Primers used were those described by Onisto and colleagues (Onisto et al, 1994) which have been described to be specific for gelatinase B. Reaction products were analysed by electrophoresis through a 2% agarose gel and visualized by ethidium bromide staining and UV transillumination.

### Northern blot analysis

Total RNA was isolated from cells as described for RT-PCR. Twenty micrograms of total cellular RNA were electrophoresed through a 1.0% agarose gel containing 6.0% formaldehyde and 1× MOPS buffer (40 mM 4-morpholinepropanesulphonic acid, 10 mM sodium acetate and 1.0 mM EDTA, pH 7.0). After electrophoresis, the separated RNAs were capillary transferred to hybond-N blotting membrane (Amersham) overnight in 10 × saline–sodium citrate (SSC). The immobilized RNAs were prehybridized at 68°C in a solution of 6 × SSC (1 × SSC is 150 mM sodium chloride (NaCl), 15 mM sodium citrate, pH 7.0), 2 × Denhardt's solution (1 × Denhardt's solution is 0.02% Ficoll (type 400), 0.02% polyvinylpyrrolidone, 0.02% BSA) and 0.5% SDS and hybridized in the same solution at 68°C with a fragment of the TIMP-1 cDNA radioactively labelled by the random priming method (Pharmacia; Quickprime kit). Hybridization was carried out overnight with agitation. After hybridization, the filter was washed twice in 2 × SSC/0.1% SDS for 15 min at 68°C and then twice in 0.1 × SSC/0.1% SDS at 68°C. The washed filter was subjected to autoradiography at − 70°C.

### Preparation of cell lysates and Western blot analysis

In the preparation of lysates, monolayers of cells were washed 3 times with cold PBS and then lysed on ice in situ in 500 µl of modified RIPA lysis buffer (1% Triton X-100 (BDH), 0.1% SDS (BDH), 50 mM Tris–HCl pH 7.5, 150 mM NaCl, 5.0 mM EDTA). After 15 min lysates were collected, centrifuged to remove debris and assayed for protein concentration by the method of Bradford (1976), as previously described. For partial purification of gelatinases, 500 µg of protein was incubated with 25 µl of gelatin immobilized on agarose beads (Sigma; 3–6 mg ml$^{-1}$) at 4°C for 2–4 h after which the beads were washed once with modified RIPA buffer and then once with PBS. Proteins bound to the gelatin were eluted with SDS-PAGE sample buffer and analysed by Western blot analysis.

 *© Cancer Research Campaign 1999*

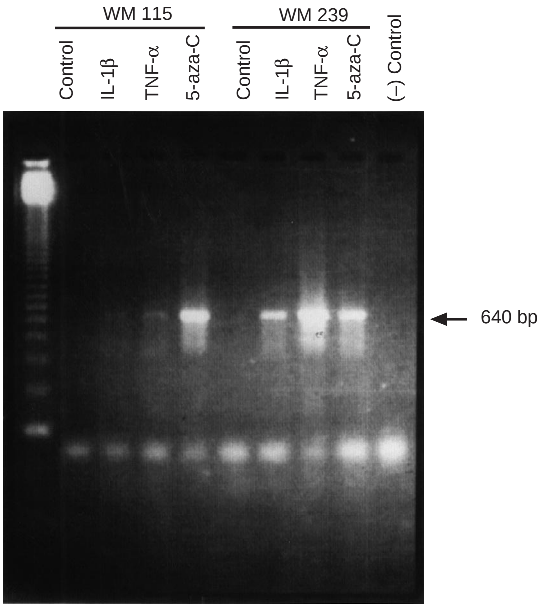

**Figure 3** Gelatinase B RT-PCR analysis of RNA isolated from melanoma cell lines. WM 115 and WM 239 cells either untreated (control) or treated with 5.0 ng ml$^{-1}$ of IL-1β, 5.0 ng ml$^{-1}$ TNF-α or 4.5 μM 2-deoxy-5-azacytidine (5-aza-c). The lane labelled MW marker is the 123 bp ladder (Gibco) and the lane labelled (–) control corresponds to the reaction in which RNA was left out of the experiment. The arrow indicates the migration of the predicted 640 bp PCR product which is indicative of the presence of gelatinase B mRNA

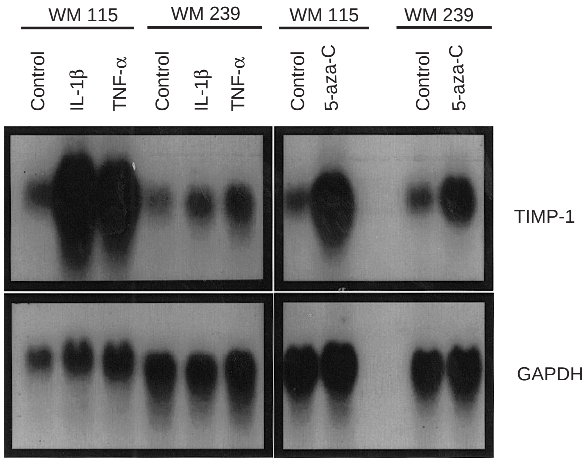

**Figure 4** TIMP-1 Northern blot analysis of RNA isolated from melanoma cell lines. WM 115 cells or WM 239 cells either untreated (control) or treated with 5.0 ng ml$^{-1}$ of IL-1β, 5.0 ng ml$^{-1}$ TNF-α or 4.5 μM 2-deoxy-5-azacytidine (5-aza-c). GAPDH hybridization is included as a control for difference in sample loading

Western blot analysis was carried out using standard techniques. Briefly, conditioned medium (10 μg of protein) that had been concentrated by ultrafiltration (Amicon microcentrifuge concentrators; 10 000 MW cut-off) or partially purified cell lysate was electrophoresed through a 10% SDS-polyacrylamide gel and electro-transferred to polyvinylidene difluoride (PVDF) blotting membrane (Immobilon-P, Millipore) overnight at 4°C in transfer buffer (50 mM Tris, 380 mM glycine, 0.1% SDS, 20% methanol) at a constant voltage of 15 V. The membrane was subsequently blocked in a solution of 5% skim milk powder in PBS/0.1% Tween-20 (PBS-T) for 1 h at ambient temperature. The membrane was probed with a murine monoclonal antibody against gelatinase B (GE 213c, a gift of Oncologix Inc., 1:1000 dilution) or affinity-purified rabbit polyclonal anti-TIMP-1 antibody (Triple Point Biologicals; 1:2500 dilution) for 2 h at ambient temperature and washed twice for 15 min each in PBS-T. The membrane was then probed with an anti-mouse or anti-rabbit horseradish peroxidase-conjugated secondary antibody for 45 min (Bio-rad, 1:5000) and similarly washed twice for 15 min each. The membrane was subsequently processed for enhanced chemiluminescence detection (ECL, Amersham) according to the manufacturer's instructions.

### Transfection and overexpression of gelatinase B cDNA

Transfection of melanoma cells either with the plasmid constructs pRc/RSV or pRc/RSV–MMP-9 were performed using Lipofectin reagent (Gibco) according to manufacturer's instructions. Briefly, 0.5–2.5 × $10^6$ cells were plated in growth media and allowed to attach overnight. The following day, 5–10 μg of purified plasmid DNA in 100 μl were mixed with an equal volume of a 1:5 dilution of lipofectin reagent and incubated at room temperature for 10 min. During this time, the monolayers of melanoma cells were washed twice with serum-free Ex-Cell 300 media. The DNA–lipofectin mixture was then diluted to a final volume of 2.0 ml and added to the cell monolayers. The cells were subsequently incubated at 37°C, 5% carbon dioxide overnight. Following this incubation the cell monolayers were washed once, re-fed with growth medium and incubated an additional 24 h prior to selection in media containing 800 μg ml$^{-1}$ of G418 (Gibco). After drug selection was complete, approximately 2 weeks, drug-resistant colonies were pooled and used for subsequent analysis.

### Assay for tumorigenicity in *nu/nu* mice

Cell lines were harvested, washed once with serum containing medium and then twice with serum-free, calcium and magnesium-free HBSS and adjusted to the appropriate concentration. Fifty microlitres of cell suspension (1.0 × $10^6$ cells for WM 239 and WM 115); 2.0 × $10^5$ for WM 239/WM 239.92 or MeWo/MeWo.92) was injected subdermally into the sides of each of four (WM 115 and WM 239) or five (WM 239/WM 239.92 or MeWo/MeWo.92) 6- to 8-week-old female *nu*/*nu* mice (Cornil et al, 1989). Tumour growth was monitored using calipers to measure two dimensions of the tumour and then applying the following formula to approximate tumour volume: volume ($cm^3$) = $width^2$ × (length/2).

### Assay for experimental metastasis formation

The same single cell suspension described for the assay of tumorigenicity was adjusted such that 200 μl of cell suspension (2.0 × $10^6$ cells, WM 239; 1.0 × $10^6$, MeWo) was injected intravenously (i.v.) into the lateral tail vein of 6 to 8-week-old female *nu*/*nu* mice. Animals were sacrificed at 10 weeks after injection of MeWo or MeWo.92 cells, or at 12 weeks after injection of WM 239 or WM 239.92 cells. At the time of sacrifice, animals were autopsied, examined for gross pathology and the lungs were fixed in Bouins fixative (BDH Chemical). The lungs were enumerated with the aid of a dissecting microscope for gross cellular colonization.

© Cancer Research Campaign 1999

### RESULTS

### Genetically related melanoma cell lines differ in their in vivo behaviour

Figure 1 depicts the resulting growth curves of WM 115 (primary melanoma) and WM 239 (metastasis) after subdermal injection of $1.0 \times 10^6$ cells. It is evident that the WM 239 tumours grow at an enhanced rate when compared to WM 115 tumours. In fact, WM 115 cells require about twice as much time (26 days) to grow to the same size (0.1 $cm^3$) as WM 239 tumours (13 days). In addition, while the WM 239 cells are able to colonize the lungs of *nu*/*nu* mice after i.v. injection, WM 115 cells are unable to do so (data not shown). Taken together, these data reflect the difference in aggressiveness of these cells and provide evidence that these two cell lines exhibit characteristics in nude mice consistent with the clinical stage at derivation.

### WM 115 and WM 239 differ in their induced expression of gelatinase B

Figure 2 shows the results of several experiments designed to examine the production of gelatinase B in WM 115 (primary melanoma) and WM 239 cells (metastasis) in response to IL-1β, TNF-α and 2-deoxy-5-azacytidine. Panel A shows, by gelatin zymography, that WM 115 cells were unable to produce gelatinase B under conditions of no treatment or with IL-1β or TNF-α stimulation. Treatment of these cells with 2-deoxy-5-azacytidine revealed some stimulation of gelatinase B production. In contrast to this, WM 239 cells were able to respond to all treatments by a marked induction of gelatinase B production. In addition, treatment of WM 239 with either TNF-α or 2-deoxy-5-azacytidine also induced the production of ‘activated’ gelatinase B as demonstrated by the presence of a band migrating slightly faster that the parent molecule. Of interest was the observation that the levels of the 72 kDa gelatinase A appeared to be unaffected by treatment with any of the agents used in these experiments. The results obtained using gelatin zymography were confirmed when the same conditioned media, or partially purified lysates from the same cells, were examined by Western blotting analysis. Figure 2B shows identical results to those found with zymographic analysis in that the enzyme protein levels in conditioned media change with cytokine or 2-deoxy-5-azacytidine treatment and similarly, as shown in Figure 2C, the same pattern of protein expression from partially purified cell lysates. Furthermore, there is also a quantitative difference in the effect that 2-deoxy-5-azacytidine has on the induction of gelatinase B in WM 115 cells when compared to WM 239 cells. Thus, it appears that in this genetically related pair of cell lines, there is a distinct difference in the ability of the cell lines to produce gelatinase B in response to the inflammatory cytokines IL-1β and TNF-α, and the DNA demethylating agent 2-deoxy-5-azacytidine, which occurs as a function of disease progression.

### TIMP-1 expression shows an opposite pattern to that of gelatinase B in WM 115 and WM 239 cells

It is known that an important inhibitor of gelatinase B, namely TIMP-1, is also modulated by the inflammatory cytokines IL-1β and TNF-α (Ito et al, 1990). With this in mind, we examined the expression of the TIMP-1 gene in this genetically related pair of cell lines in response to treatment with IL-1β, TNF-α and 2-deoxy-5-azacytidine. Figure 2D depicts Western blot analysis and demonstrates that, in contrast to the induction of gelatinase B, it is the early stage melanoma cell line, WM 115, that is responsive to cytokine and 2-deoxy-5-azacytidine treatment, whereas the advanced stage cell line WM 239, is refractory to similar cytokine treatment. As is evident from the Figure, WM 115 shows a strong induction of TIMP-1 protein levels in response to IL-1β, TNF-α and 2-deoxy-5-azacytidine while WM 239 is induced to produce TIMP-1 only with 2-deoxy-5-azacytidine treatment but is not affected by IL-1β or TNF-α treatment. Thus, it appears that while WM 115 cells respond to IL1β or TNF-α treatment by a robust up-regulation of TIMP-1 production, WM 239 cells are unable to elicit such a response.

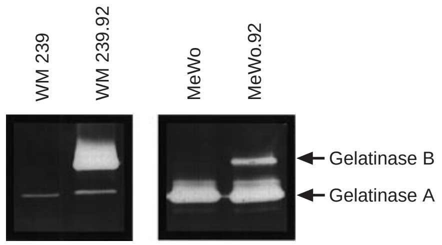

**Figure 5** Gelatin zymogram of conditioned medium derived from transfected melanoma cells. WM 239 or MeWo cells were transfected with either control or gelatinase B containing cDNA expression vectors as described in Materials and Methods. Note presence of gelatinase B activity in the cells transfected with expression vector containing gelatinase B cDNA

### Cytokine- and 2-deoxy-5-azacytidine-mediated regulation of gelatinase B and TIMP-1 occurs at the level of steady-state mRNA

We next tried to assess at what level this phenomenon was being regulated. As shown in Figure 3, the predicted 640 bp PCR product for gelatinase B, which is present in the lanes corresponding to RNA isolated from IL-1β- and TNF-α-treated WM 239 cells, is absent from the lanes corresponding to RNA isolated from similarly treated WM 115 cells. The lanes corresponding to 2-deoxy-5-azacytidine-treated WM 115 and WM 239 cells show the predicted 640 bp PCR product implying that treatment of these cells with this agent induces the transcription of gelatinase B gene in both cell lines. This assay was repeated several times on different RNA preparations and, in all cases, glyceraldehyde 3-phosphate dehydrogenase (GAPDH) was also amplified in separate reactions to ensure the integrity of the cDNA synthesis reaction (data not shown). These experiments demonstrate that the regulation of gelatinase B by IL-1β, TNF-α and 2-deoxy-5-azacytidine in these cells is most likely at the level of mRNA transcription and that the difference in the production of this molecule between these two cell lines seems to be at the level of induction of gelatinase B mRNA.

It was further determined (Figure 4) that the induction of TIMP-1 in the early-stage cell line WM 115 was at the level of the steady-state mRNA. Northern blot analysis of the same RNAs used for RT-PCR analysis depicted in Figure 3 show that, while RNAs derived from WM 115 cells show a strong induction for TIMP-1, the RNAs derived from WM 239 cells show no such induction. Thus the TIMP-1 results are a virtual mirror image of the pattern seen for gelatinase B and suggest that these two molecules may be coordinately de-regulated during the progression of human melanoma.

© Cancer Research Campaign 1999

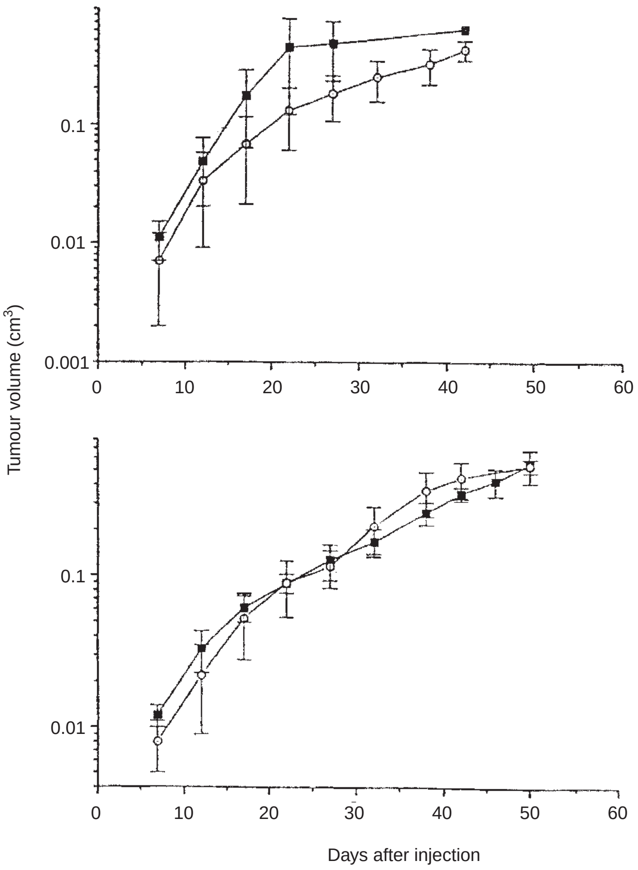

**Figure 6** Growth of control and gelatinase B-transfected melanoma cells in vivo. Two hundred thousand WM 239 (top panel) or MeWo (bottom panel) cells either transfected with control (■) or gelatinase B cDNA (⦿) containing plasmid suspended in 50 µl were injected subdermally into the flank of 6- to 8-week-old female nude mice. At the appearance of tumour growth, measurements and calculations were made as described in Materials and Methods. The data are presented as the mean ± s.d. of five animals for all groups

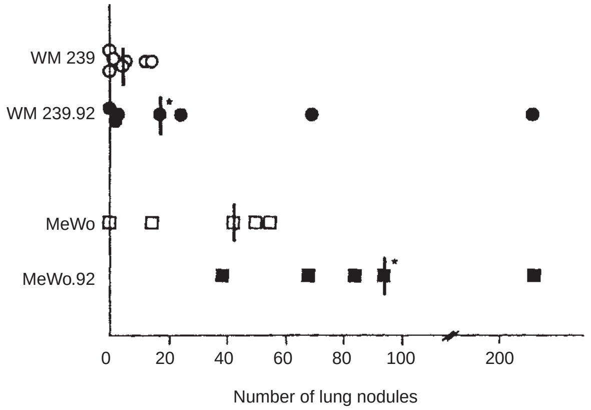

**Figure 7** Experimental metastasis assays. WM 239 (○) and WM 239.92 (●) cells ($2.0 \times 10^6$) or MeWo (□) and MeWo.92 (■) cells ($1.0 \times 10^6$) were injected into the tail vein of 6- to 8-week-old *nu/nu* mice. Mice were sacrificed 10 weeks after injection of MeWo and MeWo.92 cells or 12 weeks after injection of WM 239 and WM 239.92 cells. The results are expressed as the number of nodules for each lung examined ($n = 7$ for WM 239 and WM 239.92; $n = 5$ for MeWo and MeWo.92). The bars represent the median for each group. * represents statistical significance ($P < 0.05$) based on a non-parametric Mann–Whitney *U*-test

### Constitutive expression of gelatinase B in melanoma cells leads to an enhancement of lung colonization in vivo

Given the results so far, it was evident that there was a distinct difference in the ability of each of these cells lines to produce gelatinase B and TIMP-1 following induction with cytokines. It was also clear that, while gelatinase B was not constitutively produced by either cell line, TIMP-1 was. This prompted us to consider the effect of constitutive overexpression of gelatinase B in WM 239 cells and another melanoma cell line, MeWo, to determine the ability of these cells to colonize the lungs of *nu*/*nu* mice in an experimental metastasis assay. We surmised that if we 'tip' the proteolytic balance toward gelatinase B production over constitutive TIMP-1, there might be an enhanced tendency of the gelatinase B overexpressing cells to have an altered behaviour in vivo. Figure 5 demonstrates that cells transfected with an expression vector carrying gelatinase B cDNA overexpress this enzyme at the protein level using gelatin zymography whereas vector-transfected control cells remain gelatinase B-negative. Having derived gelatinase B overexpressing cells, we then sought to assess aspects of their phenotypic behaviour in vivo.

Figure 6 summarizes the results of tumor growth measurements of WM 239 or MeWo cells transfected with either control or gelatinase B cDNA containing vectors. For both WM 239 and WM 239.92 cells, the time for the tumours to attain a size of 0.5 $cm^3$ (approximately 0.5 g) was roughly 6 weeks with no difference in tumour take (100% for both cell lines), tumour doubling time or tumour onset. The same result was true for MeWo and MeWo.92 cells. In this case, both cell lines took about 7 weeks to reach 0.5 $cm^3$ but, similarly, showed no difference in tumour take (100% for both cell lines), tumour doubling time and no difference in tumour onset.

When WM 239, WM 239.92, MeWo and MeWo.92 cells were assessed for their ability to colonize the lungs of *nu*/*nu* mice in an experimental metastasis assay (Figure 7), it was found that the cells overexpressing gelatinase B had an enhanced ability to do so. WM 239.92 cells were found to colonize the lungs in six of seven mice injected with the median number of nodules per lung being 17 (range 0–200). The control-transfected WM 239 cells were found to colonize the lungs of only five of seven mice injected with a median number of nodules per lung being 4 (range 0–14). Two animals in the WM 239.92 group had to be sacrificed at 9 weeks due to signs of morbidity. One of these animals showed extensive tumour cell infiltration into the chest wall while the other showed signs of lower limb paralysis. A third animal, at the completion of the experiment (12 weeks), also had evidence of extensive tumour cell infiltration into the chest wall as well as a metastasis to the knee joint in a lower limb. MeWo cells overexpressing gelatinase B also showed a similar increase in the ability to colonize the lungs of *nu*/*nu* mice when compared to vector-transfected controls. MeWo.92 cells were found to colonize the lungs in five of five mice injected giving rise to a median nodule number of 84 (range 38– > 200) while control-transfected cells colonized the lungs of only four of five mice injected giving a median nodule number of 42 (range 0–55). In either case, the

© Cancer Research Campaign 1999

enhancement of lung colonization by these cells was statistically significant ($P < 0.05$) by a one-sided Mann–Whitney *U*-test.

### DISCUSSION

It is generally accepted that, while not widely expressed in normal tissues, members of the matrix metalloproteinase family of enzymes are often overexpressed in a wide variety of advanced cancers (Stetler-Stevenson et al, 1996). Furthermore, experiments involving gene transfection and overexpression of either MMPs (Powell et al, 1993; Bernhard et al, 1994) or their inhibitors (DeClerck et al, 1992; Khokha et al, 1992) into tumour cell lines or treatment of tumour-bearing animals with natural or synthetic MMP inhibitors (DeClerck et al, 1991; Chirivi et al, 1994; Wang et al, 1994) suggests a role for these molecules in tumour invasion and metastasis. Certain lines of evidence, however, suggest an alternative role for MMPs in tumour progression, that is, at the level of tumour growth (Khokha et al, 1989; Koop et al, 1994; Witty et al, 1994) and quite possibly at very early stages of benign tumour development (Wilson et al, 1997). Although the precise mechanism by which they act is at present under question, it is possible they may act through the activation and/or release of bound growth factors or receptors (Fowlkes et al, 1995; Levi et al, 1996; Manes et al, 1997; Suzuki et al, 1997). It is clear that many MMPs are overexpressed in cancers through the action of oncogenes and growth factors which represent positive or stimulatory signals for expression (Mauviel, 1993; Himelstein et al, 1997). As such, MMPs regulated in this way represent a positive 'mode' of tumour progression, that is, a gain of function is required for expression. Constitutive production of MMPs has also been linked to factors that may repress expression as demonstrated by somatic hybridization experiments (MacDougall et al, 1995). This example represents a negative or inhibitory 'mode' of tumour progression, in this case loss of function is required. In either circumstance, the expression of an MMP seems to be the consequence of a genetic 'switch' involved in tumour progression.

The pattern of regulation of endogenous MMP inhibitors, TIMPs, displays a less clear pattern of 'switching' in tumour progression. To date four TIMP family members have been characterized (TIMPs 1–4). It has been widely thought that the TIMPs are broad-spectrum inhibitors for all MMPs. There is at least one notable exception, however. Recent observations have indicated that while TIMP-2 and -3 are able to functionally inhibit MT1-MMP, TIMP-1 is not (d'Ortho et al, 1998; Will et al, 1996). The TIMPs are widely expressed in a large number of normal and neoplastic systems and with respect to some of the TIMP family members display a strong degree of tissue specific expression. For example, TIMP-3 is found tightly associated with the extracellular matrix in many tissues, while the newest member of this family, TIMP-4, is found expressed in a tissue-specific fashion in the adult mouse (Leco et al, 1997). This information, taken together, suggests that the role of metalloproteinases and their inhibitors in tumour progression is most likely at the level of MMP:TIMP balance (Liotta and Stetler-Stevenson, 1991). Given their 'positive' role in malignancy it would be predicted that in benign tumours this balance is toward MMP inhibition while in advanced, malignant cancers this balance favours proteolysis.

The data presented here suggest that melanoma cells undergo a 'switch' with respect to MMP/TIMP induction by cytokines. WM 115 cells, derived from a primary melanoma, were unable to induce gelatinase B expression in response to TNF-$\alpha$ or IL-1$\beta$ treatment while the genetically related cell line, WM 239, derived from a lymph node metastasis 16 months later, was able to do so in response to the same cytokine treatment. It could be thought that the primary cell line was, for some reason, non-responsive to TNF-$\alpha$ or IL-1$\beta$ and that sensitivity to the effects of these cytokines was a trait that the metastatic cells had acquired with progression. This, however, is not the case since WM 115 cells were able to respond to cytokine stimulation. Their response, in stark contrast to WM 239 cells, was to up-regulate the production of the metalloproteinase inhibitor, TIMP-1. To our knowledge this is the first demonstration of such a 'proteolytic switch'; a switch during tumour progression that not only involves induction of MMP production but also a loss of MMP inhibitor induction when the cells were presented with the same stimulus. Treatment of these cells with the DNA demethylating agent 2-deoxy-5-azacytidine demonstrated the ability of both cell lines to up-regulate both gelatinase B and TIMP-1. This latter effect, with respect to gelatinase B, has been subsequently linked to the induction of DNA binding proteins which complex with elements in the gelatinase B promoter (unpublished observations).

A similar situation to this has been observed with the growth inhibitory response of melanoma cells to a wide variety of cytokines, including IL-6 (Lu and Kerbel, 1993), Oncostatin M, TNF-$\alpha$ (Lu et al, 1993) and TGF-$\beta$ (MacDougall and Kerbel, 1993). These studies have found that while early-stage melanoma cells are growth inhibited by these cytokines, advanced-stage melanoma cells become resistant to the growth inhibitory action of these factors. However, what sets the present study apart from these previous studies is the fact that when confronted with the *same* stimulus both cell lines responded but in a different manner. The results presented here demonstrate a *reversal* of responsiveness to cytokine treatment as a function of tumour progression, from proteolytic inhibition to proteolytic stimulation, and not just loss of responsiveness.

The mechanism behind this observation is not at all understood. However, the data presented suggest that the cellular response to treatment, and not necessarily the treatment itself, is what governs a given pattern of gene expression. In other words, the pattern of gene expression by a given cell in response to a stimulus may not only be determined by that particular stimulus but also by what genes are *permissive* for expression under those conditions. In short, the *ability* of a cell to respond to a given factor or cytokine is as important to determination of phenotype as the agent used to elicit such a response. In the case of WM 115 and WM 239, it is clear that they are both able to respond to the same cytokine treatment; however, one cell line favours the proteolytic mode (WM 239) by up-regulation of gelatinase B while the other cell line favours the inhibitory mode (WM 115) by up-regulation of TIMP-1. This is consistent with our previous observations demonstrating that the expression of gelatinase B in 'early' vs 'advanced' melanoma cells can be controlled by a strong repressor as determined by somatic hybrid studies (MacDougall et al, 1995). This observation may, in part, explain the inability of 'early' melanoma cells to be induced to express gelatinase B. Further studies aimed at the regulation of TIMP-1 might elucidate a similar mechanism at play in this system.

Much of the current literature suggests that MMP expression in cancers is not associated with the malignant cells but in fact with stromal elements of a tumour. For example, The MMP stromelysin-3 is strongly associated of invasive breast cancers, but its expression is never associated with the tumour cells themselves

© Cancer Research Campaign 1999

(Basset et al, 1990; Chenard et al, 1996). This is true for a number of MMPs, in a variety of cancers. On the other hand, in chemically induced murine skin carcinogenesis, the MMP stromelysin-1 in fact undergoes a 'switch' in its localization of expression from stromal cells within 'early' squamous tumours, to being expressed by the tumour cells themselves in 'advanced', metastatic spindle cell tumours (Wright et al, 1994). In the case of gelatinase B it has been found that a number of aggressive cancers, when examined for expression in situ, show tumour cell-associated expression of this molecule (Pyke et al, 1992; Canete-Soler et al, 1994; Soini et al, 1994; Rao et al, 1996; Ueda et al, 1996). This information suggests that gelatinase B in particular might be an important determinant of aggressive behaviour in many cancers.

The potential role for gelatinase B in metastasis of melanoma is supported by the fact that when gelatinase B was constitutively overexpressed in two melanoma cell lines, including WM 239, an enhancement of lung colonization was observed as was the presence of extrapulmonary metastases. This is in agreement with other studies which show that overexpression of gelatinase B results in an enhancement of lung colonization (Bernhard et al, 1994) or that down-regulation of gelatinase B expression, through the action of a gelatinase B-directed ribozyme, results in a decrease in the occurrence of experimental lung metastases (Hua and Muschel, 1996; Sehgal et al, 1998). The exact mechanism by which MMP-9 acts to enhance metastasis is not well understood, however. Traditionally, it has been thought that MMPs, in general, enhance the ability of cells to invade surrounding tissues or to extravasate from the circulation (Stetler-Stevenson et al, 1993). However, recent experimental evidence suggests that MMPs may actually be operating at the level of tumour growth (Chambers and Matrisian, 1997), and gelatinase B in particular may be acting to promote angiogenesis since expression of gelatinase B has been associated with this process in a number of systems (Canete-Soler et al, 1995; Vu et al, 1998). It is interesting to speculate on the possible in vivo role for the observed 'switch' in cytokine responsiveness in melanoma progression. It is known, somewhat paradoxically, that a brisk host inflammatory response leading to regression of the lesion is, in fact, a poor prognostic factor for the development of metastases in patients with melanoma (Gromet et al, 1978; Ronan et al, 1987; Clark et al, 1989). Given the role of TNF-α and IL-1β in such an inflammatory response it is quite possible that this 'switch' in responsiveness to inflammatory cytokines might represent a molecular basis for the selection of aggressive variants, or the onset of angiogenesis, during such a host response which, in turn, portends to an unfavourable disease outcome.

### ACKNOWLEDGEMENTS

This work was supported by grant CA 41233 from the National Institutes of Health (NIH), USA to RSK and from the Italian Association for Cancer Research (AIRC) to MRB. RSK is a Terry Fox Research Scientist of the National Cancer Institute of Canada. JRM was supported by a studentship from the Medical Research Council of Canada. We thank Dr Meenard Herlyn, Wistar Institute, Philadelphia for the WM melanoma cell lines used in our studies.

### REFERENCES

Basset P, Belloq JP, Wolf C, Stoll I, Hutin P, Limacher JM, Podhajcer OL, Chenard MP, Rio MC and Chambon P (1990) A novel metalloproteinase gene specifically expressed in stromal cells of breast carcinomas. *Nature* **348**: 699–704

Bernhard EJ, Gruber SB and Muschel RJ (1994) Direct evidence linking expression of matrix metalloproteinase 9 (92-kDa gelatinase/ collagenase) to the metastatic phenotype in transformed rat embryo cells. *Proc Nat Acad Sci USA* **91**: 4293–4297

Bradford MM (1976) A rapid and sensitive method for the quantitation of microgram quantities of protein utilizing the principle of protein-dye binding. *Analyst Biochem* **72**: 248–254

Canete-Soler R, Litzky L, Lubensky I and Muschel RJ (1994) Localization of the 92 kD gelatinase mRNA in squamous cell and adenocarcinomas of the lung using in situ hybridization. *Am J Pathol* **144**: 518–527

Canete-Soler R, Gui YH, Linask KK and Muschel RJ (1995) MMP-9 (gelatinase B) mRNA is expressed during mouse neurogenesis and may be associated with vascularization. *Brain Res* **88**: 37–52

Chambers AF and Matrisian LM (1997) Changing views of the role of matrix metalloproteinases in metastasis. *J Nat Cancer Inst* **89**: 1260–1270

Chenard MP, Osiorain L, Shering S, Rouyer N, Lutz Y, Wolf C, Basset P, Bellocq JP and Duffy MJ (1996) High levels of stromelysin-3 correlate with poor prognosis in patients with breast carcinoma. *Int J Cancer* **69**: 448–451

Chirivi RGS, Garofalo A, Crimmin MJ, Bawden LJ, Stoppacciaro A, Brown PD and Giavazzi R (1994) Inhibition of the metastatic spread and growth of B16-BL6 murine melanoma by a synthetic matrix metalloproteinase inhibitor. *Int J Cancer* **58**: 460–464

Chomczynski P and Sacchi N (1987) Single-step method of RNA isolation by acid guanidinium thiocyanate-phenol-chloroform extraction. *Analyt Biochem* **162**: 156–159

Clark WH, Elder DE, Guerry D, Braitman LE, Trock BJ, Schultz D, Synnestvedt M and Halpern AC (1989) Model predicting survival in stage I melanoma based on tumor progression. *J Nat Cancer Inst* **81**: 1983–1904

Collier IE, Wilhelm SM, Eisen AZ, Marmer BL, Grant GA, Seltzer JL, Kronberger A, He C, Bauer EA and Goldberg GI (1988) H-ras oncogene-transformed human bronchial epithelial cells (TBE-1) secrete a single metalloproteinase capable of degrading basement membrane collagen. *J Biol Chem* **263**: 6579–6587

Cornil I, Man S, Fernandez B and Kerbel RS (1989) Enhanced tumorigenicity, melanogenesis, and metastases of a human malignant melanoma after subdermal implantation in nude mice. *J Natl Cancer Inst* **81**: 938–944

DeClerck YA and Imren S (1994) Protease inhibitors: role and potential therapeutic use in human cancer. *Eur J Cancer* **30A**: 2170–2180

DeClerck YA, Yean T-D, Chan D, Shimada H and Langley KE (1991) Inhibition of tumor invasion of smooth muscle cell layers by recombinant human metalloproteinase inhibitor. *Cancer Res* **51**: 2151–2157

DeClerck YA, Perez N, Shimada H, Boone TC, Langley KE and Taylor SM (1992) Inhibition of invasion and metastasis in cells transfected with an inhibitor of metalloproteinases. *Cancer Res* **52**: 701–708

d'Ortho MP, Stanton H, Butler M, Atkinson SJ, Murphy G and Hembry RM (1998) MT1-MMP on the cell surface causes focal degradation of gelatin films. *FEBS Lett* **421**: 159–164

Edwards DR, Murphy G, Reynolds JJ, Whitham SE, Docherty AJP, Angle P and Heath JK (1987) Transforming growth factor-β modulates the expression of collagenase and metalloproteinase inhibitor. *EMBO J* **6**: 1899–1904

Fowlkes JL, Thrailkill KM, Serra DM, Suzuki K and Nagase H (1995) Matrix metalloproteinases as insulin-like growth factor binding protein-degrading proteinases. *Prog Growth Factor Res* **6**: 255–263

Frisch SM, Reich R, Collier IE, Genrich LT, Martin G and Goldberg GI (1990) Adenovirus E1A represses protease gene expression and inhibits metastasis of human tumor cells. *Oncogene* **5**: 75–83

Herron GS, Banda MJ, Clark EJ, Gavrilovic J and Werb Z (1986) Secretion of metalloproteinases by stimulated capillary endothelial cells. **261**: 2814–2818

Himelstein BP, Lee EJ, Sato H, Seiki M and Muschel RJ (1997) Transcriptional activation of the matrix metalloproteinase 9 gene in an h-*ras* and v-*myc* transformed rat embryo cell line. *Oncogene* **14**: 1995–1998

Hua J and Muschel RJ (1996) Inhibition of matrix metalloproteinase 9 expression by a ribozyme blocks metastasis in a rat sarcoma model system. *Cancer Res* **56**: 5279–5284

Ito A, Sato T, Iga T and Mori Y (1990) Tumor necrosis factor bifunctionally regulates matrix metalloproteinases and tissue inhibitor of metalloproteinases (TIMP) production by human fibroblasts. *FEBS Lett* **269**: 93–95

Kath R, Rodeck U, Parmiter A and Herlyn M (1991) Growth factor independence in vitro of primary melanoma cells from advanced but not early or intermediate lesions. *Cancer Therapy Control* **1**: 179–191

Khokha R, Waterhouse P, Yagel S, Lala PK, Overall CM, Norton G and Denhardt DT (1989) Antisense RNA-induced reduction in murine TIMP levels confers oncogenicity on Swiss 3T3 cells. *Science* **243**: 947–950

© Cancer Research Campaign 1999

Khokha R, Zimmer MJ, Graham CH, Lala PK and Waterhouse P (1992) Suppression of invasion by inducible expression of tissue inhibitor of metalloproteinase-1 (TIMP-1) in B16-F10 melanoma cells. *J Nat Cancer Inst* **84**: 1017–1022

Koop S, Khokha R, Schmidt EE, MacDonald IC, Morris VL, Chambers AF and Groom, AC (1994) Overexpression of metalloproteinase inhibitor in B16F10 cells does not affect extravasation but reduces tumor growth. *Cancer Res* **54**: 4791–4797

Leco KJ, Apte SS, Taniguchi GT, Hawkes SP, Khokha R, Schultz GA and Edwards DR (1997) Murine tissue inhibitor of metalloproteinases-4 (TIMP-4): cDNA isolation and expression in adult mouse tissue. *FEBS Lett* **401**: 213–217

Levi E, Fridman R, Miao HQ, Ma YS, Yayon A and Vlodavsky I (1996) Matrix matalloproteinase 2 releases active soluble ectodomain of fibroblast growth factor receptor 1. *Proc Natl Acad Science USA* **93**: 7069–7074

Librach CL, Werb Z, Fitzgerald ML, Chiu K, Corwin NM, Esteves RA, Grobelny D, Galardy R, Damsky CH and Fisher SJ (1991) 92-kD type IV collagenase mediates invasion of human cytotrophoblasts. *J Cell Biol* **113**: 437–449

Liotta LA and Stetler-Stevenson WG (1991) Tumor invasion and metastasis: an imbalance of positive and negative regulation. *Cancer Res* **51**: 5054s–5059s

Lu C and Kerbel RS (1993) Interleukin-6 undergoes transition from paracrine growth inhibitor to autocrine stimulator during human melanoma progression. *J Cell Biol* **120**: 1281–1288

Lu C, Rak JW, Kobayashi H and Kerbel RS (1993) Increased resistance to oncostatin M-induced growth inhibition of human melanoma cell lines derived from advanced-stage lesions. *Cancer Res* **53**: 2708–2711

MacDougall JR and Kerbel RS (1993) Responsiveness of normal/dysplastic melanocytes and melanoma cells from different lesional stages of disease progression to the growth inhibitory effects of TGF-β. *Mol Cell Diff* **1**: 21–40

MacDougall JR and Matrisian LM (1995) Contributions of tumor and stromal matrix metalloproteinases to tumor progression, invasion and metastasis. *Cancer Metastasis Rev* **14**: 351–362

MacDougall JR, Bani MR, Lin Y, Rak J and Kerbel RS (1995) The 92 kDa gelatinase B is expressed by advanced stage melanoma cells: suppression by somatic cell hybridization with early stage melanoma cells. *Cancer Res* **55**: 4174–4181

Manes S, Mira E, Barbacid MM, Cipres A, Fernandez-Resa P, Buesa JM, Merida I, Aracil, M, Marquez G and Martinez AC (1997) Identification of insulin-like growth factor-binding protein-1 as a potential physiological substrate for human stromelysin-3. *J Biol Chem* **272**: 25706–25712

Mauviel A (1993) Cytokine regulation of metalloproteinase gene expression. *J Cell Biochem* **53**: 288–295

Moll UM, Youngleiv GL, Rosinski KB and Quigley JP (1990) Tumor promoter-stimulated $M_r$ 92,000 gelatinase secreted by normal and malignant human cells: isolation and characterization of the enzyme from HT1080 tumor cells. *Cancer Res* **50**: 6162–6170

Mustoe TA, Pierce GF, Thomason A, Gramates P, Sporn MB and Deuel TF (1987) Accelerated healing of incisional wounds in rats induced by transforming growth factor-beta. *Science* **237**: 1333–1336

Onisto M, Garbisa S, Caenazzo C, Freda MP, DiFrancesco C, Nitti D, Liotta LA and Stetler-Stevenson WG (1994) Reverse transcription-polymerase chain reaction phenotyping of metalloproteinase and inhibitors involved in tumor matrix invasion. *Diagn Mol Pathol* **2**: 74–80

Powell WC, Knox JD, Navre M, Grogan TM, Kittelson J, Nagle RB and Bowden GT (1993) Expression of the metalloproteinase matrilysin in Du-145 cells increases their invasive potential in severe combined immunodeficient mice. *Cancer Res* **53**: 417–422

Pyke C, Ralfkiaer E, Huhtala P, Hurskainen T, Dano K and Tryggvason K (1992) Localization of messenger RNA for $M_r$ 72,000 and 92,000 type IV collagenases in human skin cancers by in situ hybridization. *Cancer Res* **52**: 1336–1341

Rao JS, Yamamoto M, Mohaman S, Gokaslan ZL, Fuller GN, Stetler-Stevenson WG, Rao VH, Liotta LA, Nicolson GL and Sawaya RE (1996) Expression and localization of 92 kDa type IV collagenase gelatinase B (MMP-9) in human gliomas. *Clin Exp Metastasis* **14**: 12–18

Ronan SG, Eng AM, Briele HA, Shioura NN and Das Gupta TK (1987) Thin malignant melanomas with regression and metastases. *Arch Dermatol* **123**: 1326–1330

Salo T, Lyons JG, Rahemtulla F, Birkedal-Hansen H and Larjava H (1991) Transforming growth factor-beta1 up-regulates type IV collagenase expression in cultured human keratinocytes. *J Biol Chem* **266**: 11436–11441

Sato H and Seiki M (1993) Regulatory mechanisms of 92 kDA type IV collagenase gene expression which is associated with invasiveness of tumor cells. *Oncogene* **8**: 395–405

Sehgal G, Hua J, Bernhard EJ, Sehgal I, Thompson TC and Muschel RJ (1998) Requirement for matrix metalloproteinase 9 (gelatinase B) expression in metastasis by murine prostate carcinoma. *Am J Pathol* **152**: 591–596

Soini Y, Hurskainen T, Hoyhtya M, Oikarinen A and Autio-Harmainen H (1994) 72 kD and 92 kD type IV collagenase, type IV collagen, and laminin mRNAs in breast cancer: a study by in situ hybridization. *J Histochem Cytochem* **42**: 945–951

Stetler-Stevenson WG, Liotta LA and Kleiner DE (1993) Extracellular matrix 6: ole of matrix metalloproteinases in tumor invasion and metastasis. *FASEB J* **7**: 1434–1441

Stetler-Stevenson WG, Hewitt R and Corcoran M (1996) Matrix metalloproteinases and tumor invasion – from correlation and causality to the clinic. *Semin Cancer Biol* **7**: 147–154

Suzuki M, Raab G, Moses MA, Fernandez CA and Klagsbrun M (1997) Matrix metalloproteinase-3 releases active heparin-binding EGF-like growth factor by cleavage at a specific juxtamembrane site. *J Biol Chem* **272**: 31730–31737

Ueda Y, Imai K, Tsuchiya H, Fujimoto N, Nakanishi I, Katsuda S, Seiki M and Okada Y (1996) Matrix metalloproteinase 9 (gelatinase B) is expressed in multinucleated giant cells of human giant cell tumor of bone and is associated with vascular invasion. *American J Pathol* **148**: 611–622

Unemori EN, Hibbs MS and Amento EP (1991) Constitutive expression of a 92-kD gelatinase (type V collagenase) by rheumatoid synovial fibroblasts and its induction in normal human fibroblasts by inflammatory cytokines. *J Clin Invest* **88**: 1656–1662

Vu TH, Shipley JM, Bergers G, Berger JE, Helms JA, Hanahan D, Shapiro SD, Senior RM and Werb Z (1998) MMP-9/gelatinase B is a key regulator of growth plate angiogenesis and apoptosis of hypertrophic chondrocytes. *Cell* **93**: 411–422

Wang X, Fu X, Brown PD, Crimmin MJ and Hoffman RM (1994) Matrix metalloproteinase inhibitor BB-94 (batimastat) inhibits human colon tumor growth and spread in a patient-like orthotopic model in nude mice. *Cancer Res* **54**: 4726–4728

Watanabe H, Nakanishi I, Yamashita K, Hayakawa T and Okada Y (1993) Matrix metalloproteinase-9 (92 kDa gelatinase/type IV collagenase) from U937 monoblastoid cells: correlation with cellular invasion. *J Cell Sci* **104**: 991–999

Wilhelm SM, Collier IE, Marmer BL, Eisen AZ, Grant GA and Goldberg GI (1989) SV-40 transformed human lung fibroblasts secrete a 92-kDa type IV collagenase which is identical to that secreted by normal human macrophages. *J Biol Chem* **264**: 17213–17221

Will H, Atkinson SJ, Butler GS, Smith B and Murphy G (1996) The soluble catalytic domain of membrane type 1 matrix metalloproteinase cleaves the propeptide of progelatinase A and initiates autoproteolytic activation. Regulation by TIMP-2 and TIMP-3. *J Biol Chem* **271**: 17119–17123

Wilson CL, Heppner KJ, Labosky PA, Hogan BLM and Matrisian LM (1997) Intestinal tumorigenesis is suppressed in mice lacking the metalloproteinase matrilysin. *Proc Natl Acad Sci USA* **94**: 1402–1407

Witty JP, McDonnell S, Newell KJ, Cannon P, Navre M, Tressler RJ and Matrisian LM (1994) Modulation of matryilysin levels in colon carcinoma cell lines affects tumorigenicity in vivo. *Cancer Res* **54**: 4805–4812

Wright JH, McDonnell S, Portella G, Bowden GT, Balmain A and Matrisian LM (1994) A switch from stromal to tumor cell expression of stromelysin-1 mRNA associated with the conversion of squamous to spindle carcinomas during mouse skin tumor progression. *Mol Carcinogen* **10**: 207–215

 *© Cancer Research Campaign 1999*
